# Fluorescence Turn-Off Ligand for Parallel G-Quadruplexes

**DOI:** 10.3390/molecules29163907

**Published:** 2024-08-18

**Authors:** Joanna Nowak-Karnowska, Agata Głuszyńska, Joanna Kosman, Anna Dembska

**Affiliations:** 1 Department of Bioanalytical Chemistry, Faculty of Chemistry, Adam Mickiewicz University, Uniwersytetu Poznańskiego 8, 61-614 Poznań, Poland; j.nowak@amu.edu.pl (J.N.-K.); aglusz@amu.edu.pl (A.G.);; 2 Laboratory of Molecular Assays and Imaging, Institute of Bioorganic Chemistry, Polish Academy of Sciences, Noskowskiego 12/14, 61-704 Poznań, Poland

**Keywords:** parallel G-quadruplexes, 9-methoxyluminarine, fluorescence, CD spectroscopy, UV–vis spectroscopy

## Abstract

Parallel-stranded G-quadruplex structures are found to be common in the human promoter sequences. We tested highly fluorescent 9-methoxyluminarine ligand (9-MeLM) binding interactions with different parallel G-quadruplexes DNA by spectroscopic methods such as fluorescence and circular dichroism (CD) titration as well as UV melting profiles. The results showed that the studied 9-MeLM ligand interacted with the intramolecular parallel G-quadruplexes (G4s) with similar affinity. The binding constants of 9-methoxyluminarine with different parallel G4s were determined. The studies upon oligonucleotides with different flanking sequences on c-*MYC* G-quadruplex suggest that 9-methoxyluminarine may preferentially interact with 3′end of the c-*MYC* promoter. The high decrease in 9-MeLM ligand fluorescence upon binding to all tested G4s indicates that 9-methoxyluminarine molecule can be used as a selective fluorescence turn-off probe for parallel G-quadruplexes.

## 1. Introduction

Guanine quadruplexes (G-quadruplex DNA, G4 DNA) are forms of DNA, important in the research on cancer and telomerase, as well as in the research on transcriptional changes by the formation of G-quadruplexes in the promoter region of genes [1,2]. These non-canonical single- or multistranded nucleic acid structures are formed by co-planar arrangements of four guanines (G-tetrads), stabilized by Hoogsteen-type hydrogen bonds in the presence of selected metal cations (Na^+^ and K^+^) and can be stabilized by small organic ligands. Until now, many organic compounds that induce, stabilize or disrupt the structure of G4 have been obtained and are collected in the updated G4 and i-motifs ligand database, G4LDB 2.2 [3]. Ligands with negligible affinity for DNA duplexes and ligands with specificity for some G4s over others are still being sought [4,5,6]. However, some selective ligands have been recently identified such as a thiazole peptide [7], pyridinium salt [8], a coumarin–quinazolinone [9], a quinazoline–quinazolinone [10], a core-extended naphthalene diimide [11], squaraine dyes [12,13], and anthracene-based [14], BODIPY-based [15], triarylimidazole-based [16], cyanine dye-based [17] and bis(quinolinium) pyridodicarboxamide-based [18] compounds.

Fluorescent ligands interacting with G4 can be classified as follows: (1) “light-up” probes that display a strong enhancement upon G4 binding, (2) “light-off” probes that display a decreased fluorescence upon G4 binding and (3) permanent probes (“tagged” G4-binders) that exhibit no changes in fluorescence signals but possess high quadruplex binding specificity [19]. For instance, “light-off” probes are in general less suitable for imaging in vivo, whereas they may be very useful for monitoring interactions and evaluating binding affinity and selectivity for G-quadruplexes.

A small ligand, 1-amino-9-methoxy-2,4,10-triaza-4b-azoniaphenanthrene (9-methoxyluminarine, 9-MeLM), has a planar heteroaromatic core, a structural element characteristic of G-quadruplex ligands, as well as very favorable photophysical properties, such as solubility (is positively charged), a high fluorescence quantum yield (0.99), a luminescence lifetime of 10 ns and photostability [20].

**Figure 1:**
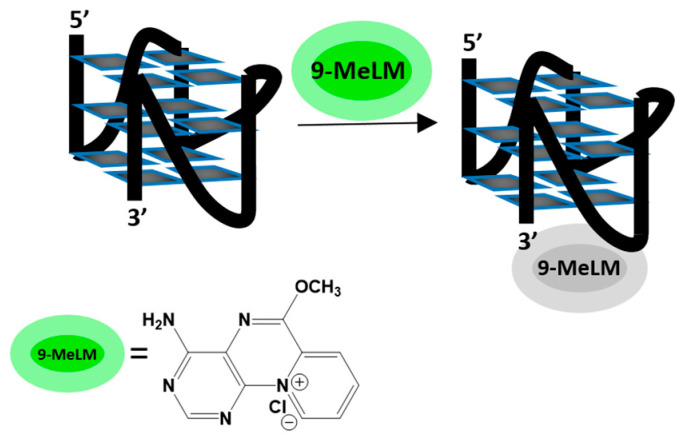
A schematic illustration of binding mode between 9-MeLM ligand and c-*MYC* G-quadruplex.

Previously, we have shown that the fluorescence of the 9-methoxyluminarine (9-MeLM) ligand is selectively and effectively quenched by the c-*MYC* G-quadruplex over nonparallel G4s. We proved that observed quenching occurs in a static mode, most probably due to a π-π interaction between 9-MeLM and the exposed parallel G4 guanine tetrad (Figure 1) [21]. In the case of parallel G-quadruplexes, it was shown that the terminal-stacking binding mode of selective ligands is the main kind of interaction for them, which was supported by molecular docking and mechanistic studies [22]. Lately, Deiana et al. developed a fluorescent probe with a minimalistic amidinocoumarin-based molecular scaffold that selectively recognizes parallel G4 structures, which is manifested by turn-on emission [23]. In their case, titration of the probe with c-*MYC* sG4 (16-mer being only c-*MYC* G4 core) induced 6-fold higher enhancement in fluorescence signals than for complexation with 22-mer c-*MYC* (possessing TGA at the 5′ end and TAA at the 3′ end). Hence, they indicated that its light-up ability was higher and correlated with better π-stacking possibilities for the planar conformation of the coumarin core [23]. On the other hand, the studies by Gai et al. indicated that the flanking sequence might form a binding cavity above the terminal G-quartet for ligands and interact with ligands through π-π stacking or, in the opposite manner, exhibit steric hindrance effects to hamper ligand/G4 binding [24]. Their results revealed that the flanking sequences provide a positive facilitating effect when the flanking chain is extended to two to five residues, whereas a negative hindrance effect to ligand binding was caused by one or six residues (the overlong flanking sequence) [24]. 

Since our previous work suggested selectivity of 9-MeLM for parallel G4s over nonparallel ones, we decided to check the binding affinity of highly fluorescent 9-MeLM to other parallel G4s. For this purpose, we have examined interaction of the 9-methoxyluminarine ligand with G-quadruplexes formed by sequences that correspond to the human proto-oncogenes c-*KIT* and *RET*, as well as hemin aptamer catG4, well known to form parallel topology. The c-*KIT* proto-oncogene encodes a receptor tyrosine kinase that is involved in the regulation of a number of physiological processes such as proliferation, differentiation, migration, maturation and cell survival and the overexpression or mutation of the c-*KIT* proto-oncogene that is identified in many diseases [25,26,27]. As a research template, we chose a well-characterized 22-mer G-rich sequence derived from c-*KIT1* that forms unique parallel-stranded intramolecular G-quadruplex (PDB ID 2O3M) [28]. Another proto-oncogene that also encodes a receptor tyrosine kinase is the proto-oncogene *RET*, which is expressed in tissues and tumors, and its activation contributes to the development of human cancers [29,30]. We chose a 20-mer sequence derived from *RET* as the molecular target in our studies (PDB ID 2L88) [31]. The hemin-binding DNA aptamer catG4 is commonly used to obtain DNAzymes [32,33]. In our experiments, intramolecular parallel G4-forming sequence of catG4 was used as model target [32].

Therefore, we present the stability and interaction of 9-methoxyluminarine ligand (9-MeLM) with various parallel G-quadruplexes by spectroscopic methods such as fluorescence and circular dichroism (CD) titration and UV melting profiles. Moreover, a few sequences without 3′ or 5′ flanking sequence extension based on c-*MYC* were designed to gain insight into the (c-*MYC* G-quadruplex)/9-MeLM ligand interaction. We demonstrate the possibility of using the 9-methoxyluminarine molecule and the common fluorescence spectroscopy technique as a preliminary screening allowing the recognition of parallel G-quadruplex topology. 

## 2. Results and Discussion

### 2.1. Circular Dichroism Spectroscopy

**Figure 2:**
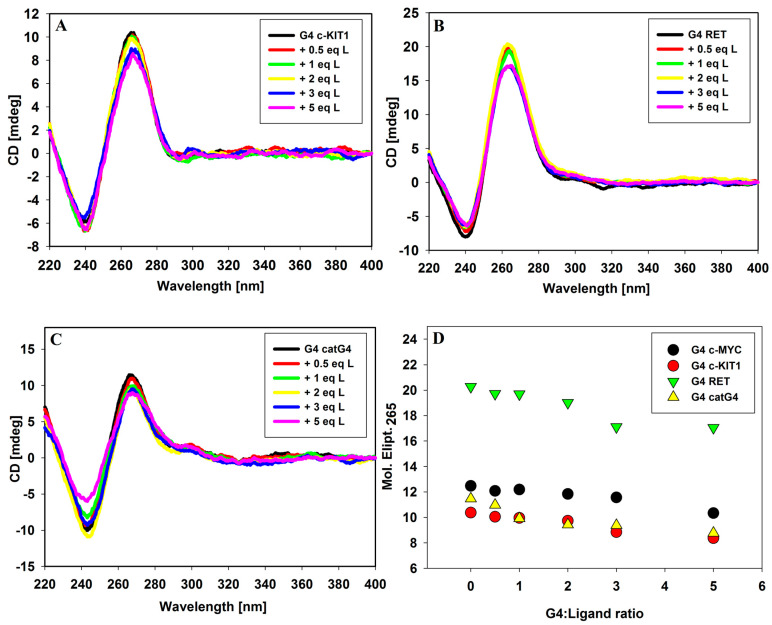
Circular dichroism (CD) spectra of G-quadruplexes c-*KIT1* (**A**), *RET* (**B**) and catG4 (**C**) with increasing amounts of 9-methoxyluminarine ligand; (**D**) CD signal changes at 265 nm against G-quadruplexes DNA/ligand molar ratio. Conditions: 10 mM Tris-HCl buffer (pH 7.2), 100 mM KCl, [G4] = 5 µM.

Circular dichroism spectroscopy (CD) has become a very useful technique in the study of G-quadruplex structure and of G-quadruplex/ligand interactions. By analyzing CD spectra, we can study the influence of various factors such as, e.g., the pH, temperature, type and concentration of ions or the presence of ligands on conformational changes in DNA. In the short-wavelength region where DNA possess absorption bands, the CD bands appear, whose position and magnitude are commonly assigned to specific G-quadruplex conformations. A typical parallel G-quadruplex structure exhibit a positive CD signal at about 265 nm and a negative CD signal at 240 nm in the presence of 100 mM KCl [34]. Thus, CD spectroscopy can be used to effectively distinguish between parallel and antiparallel G-quadruplex structures [35]. Circular dichroism spectra were recorded to assess the effect of the 9-methoxyluminarine ligand on the conformation of all tested parallel G4s, to monitor stability and to verify the binding mode in the ligand/G4 complex. Upon addition of the ligand, the intensity of peaks changed slightly (between 15% and 25% at DNA/ligand molar ratio, 1:5), but their position was stable, suggesting that the general topology remains intact (Figure 2). The corresponding UV–vis spectra are shown in Supplementary Materials (Figure S1). The induced signals (ICD) did not occur in the long-wavelength range, where absorption band with maximum at 390 nm of achiral 9-methoxyluminarine is present. Based on this observation, we can exclude the groove binding to G-quadruplex structures, because a positive ICD, signal as well as the induced exciton CD signal have been taken as an indicator of groove binding to G-quadruplex structures [36]. These results indicated that the G-quadruplexes formed by c-*KIT*1, *RET* and catG4 bind tested ligand similarly to c-*MYC* G4 [21]. The slight decrease in the intensity of the CD at 265 nm may be caused by the interaction of chromoforic group of ligand and outer G-tetrads in the parallel G-quadruplexes [37,38,39]. Finally, the most likely binding mode of 9-methoxyluminarine to the parallel G-quadruplexes’ DNA is via end-stacking interactions with external G-tetrads, which are easily accessible (tested G4s possess only sidewise loops).

**Figure 3:**
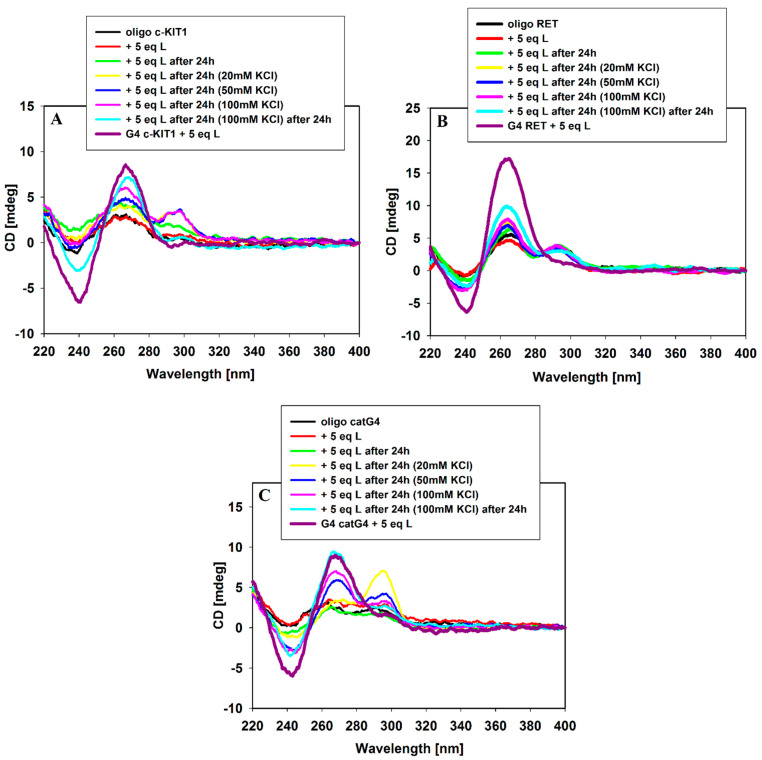
CD spectra of c-*KIT1* (**A**), *RET* (**B**), catG4 (**C**) and oligonucleotides (5 µM) with 5 equiv. of 9-methoxyluminarine ligand in Tris–HCl buffer (10 mM, pH 7.2) and increasing amounts (0–100 mM) of KCl.

Moreover, we used this analytical technique to check whether 9-methoxyluminarine has the ability to induce the G-quadruplex folding without the addition of potassium ions. The 9-methoxyluminarine ligand is not able to induce the folding of single-stranded G-rich DNA into quadruplex structures in the absence of K^+^ even after 24 h (Figure 3) as we expected on the basis on our previous studies [21]. DNA oligonucleotide conformational changes were clearly observed after the addition of 20, 50 and 100 mM KCl, and even after 24 h from these additions. The smallest effect was observed in the case of the *RET* oligonucleotide after the addition of 100 mM KCl; however, after 24 h, a slight increase in the intensity of the positive signal at 265 nm was noticed. On the other hand, all CD spectra of *RET* oligonucleotide (regardless of the compounds added) exhibit the presence of a very weak positive band at 295 nm, which could indicate the existence of a small portion of the antiparallel structure in the tested mixture. In the case of catG4 oligonucleotide, the addition of 20 mM KCl caused the formation of a mixed parallel-/antiparallel-stranded structure, predominantly as an antiparallel structure as evidenced by the major positive signal which appeared at 295 nm. The same addition of potassium ions resulted in a mixture of parallel and antiparallel G4s for the c-*KIT1* oligonucleotide. After the addition of 100 mM KCl to c-*KIT1* or catG4 oligonucleotides, their CD spectra were dominated by the parallel over the antiparallel G4 form. After 24 h, only parallel G-quadruplexes were observed (Figure 3). These results suggest that the formation of parallel G-quadruplexes in the presence of the 9-MeLM ligand, but induced by potassium ions, occurs with the participation of the antiparallel form. Moreover, we could observe that the kinetics of final folding into parallel G4s is different for each tested sequence.

### 2.2. DNA Melting Studies

**Figure 4:**
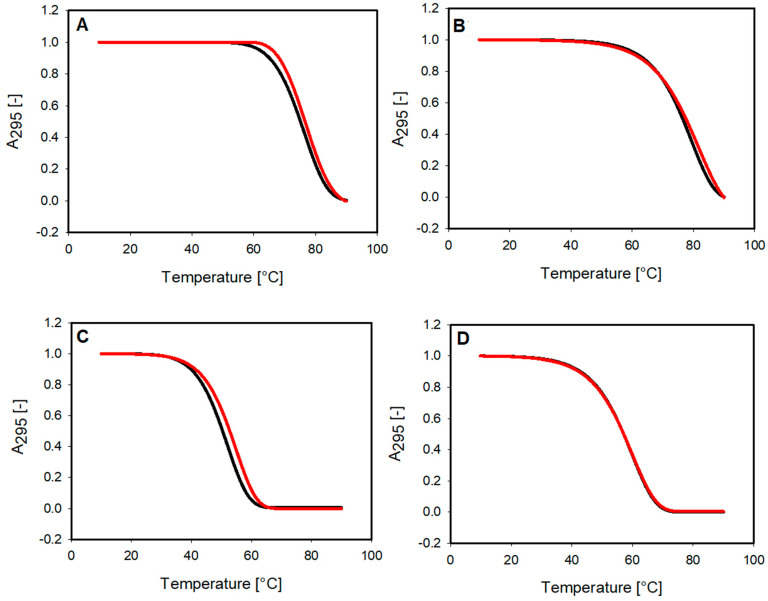
Normalized UV melting profiles (melting curves) of 2 μM G-quadruplexes (black), with and without 3 equiv. of 9-MeLM ligand (red) in 10 mM Tris–HCl buffer (pH 7.2) containing 10 mM KCl/90 mM LiCl (**A**) *RET*, (**B**) c-*MYC*, (**C**) c-*KIT1* and (**D**) catG4.

We performed UV-monitored melting experiments to investigate the thermodynamic properties of G-quadruplexes and their complexes with the small molecule of 9-methoxyluminarine. Our thermal denaturation studies were carried out in 10 mM Tris–HCl buffer (pH 7.2) containing the stabilizing potassium cation in a concentration reduced to 10 mM and with the addition of 90 mM LiCl. Thanks to this approach, the proper melting range could be observed, and the effect of the ligand could be evaluated. The melting curves of G-quadruplexes were found to be monophasic, and the same effect was observed for curves obtained for G4 with the presence of three equiv. of the ligand (Figure 4). Temperatures at half transition for the studied complexes can be found in Table 1. The obtained T_m_ indicates that the 9-MeLM ligand does not significantly affect the stability of the G-quadruplexes with parallel topology. The hysteresis was observed between the melting and annealing curves (temperature change rate of 1 °C per minute), which indicates the slow kinetics of the processes (Figure S2) [40,41].

### 2.3. Binding Parameters via Fluorescence Spectroscopy

**Figure 5:**
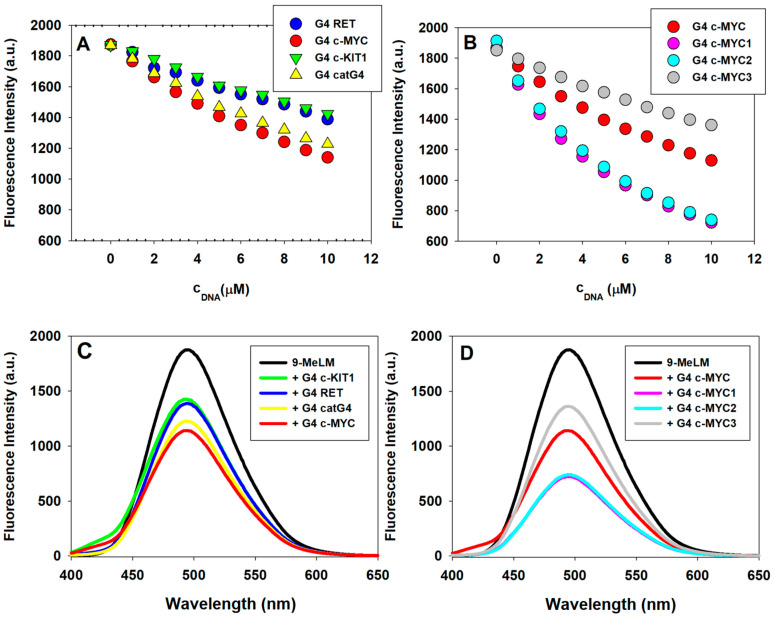
The fluorescence intensity (at 495 nm, λ_ex_ 390 nm) of 9-methoxyluminarine (9-MeLM) vs. the increasing concentration of G-quadruplexes (G4s) (**A**,**B**) and fluorescence spectra recorded for 9-methoxyluminarine in Tris–HCl buffer (10 mM, pH 7.2), KCl (100 mM) w/o (black) and with 10 equiv. of different G4s (**C**,**D**).

9-Methoxyluminarine (9-MeLM) emits bright and stable fluorescence in the visible region with its maximum at 495 nm. We used fluorescence titration method to obtain the binding characterization of 9-MeLM to the studied G4s, such as *RET*, c-*MYC*, c-*KIT1*, catG4, c-*MYC1*, c-*MYC2*, and c-*MYC3*. Compared with c-*MYC1* (being indeed core of c-*MYC*), c-*MYC2* has a 5′-end flanking sequence TAA, c-*MYC3* has a 3′-end flanking sequence TAA while c-*MYC* has two flanking sequences on both ends (TGA at 5′-end and TAA at 3′-end). As in our previous work [21], the quenching of 9-MeLM was observed during the addition of chosen G-quadruplexes. A decrease in the fluorescence intensity at 495 nm with increasing concentration of G4 (1–10 μM) indicates its binding to the G-quadruplex structure (Figure 5 and Figure S3). As shown in Figure 5B, the changes in 9-MeLM fluorescence intensity under the same c-*MYC* G4 to 9-MeLM ratio, indicating the order of binding affinity to 9-MeLM, is c-*MYC3* (with 3′-end flanking sequence) < c-*MYC* (with both 5′-end and 3′-end flanking sequences) < c-*MYC2* (with the 5′-end flanking sequence) < c-*MYC1* (without flanking sequences at both ends). 

The fluorescence data were analyzed using the Stern–Volmer equation [42]:

F_0_/F =1 + K_SV_ [Q] (1)

where K_SV_ is the Stern–Volmer constant, Q is the total quencher concentration, and F and F_0_ are the fluorescence intensities in the presence and absence of the quencher (here, G4), respectively. In all cases, we observed a linear relationship between 9-MeLM fluorescence and G4 concentration, with a correlation coefficient higher than 0.990 (Figures S4 and S5). It is worth mentioning that we have recently proved that the fluorescence of the 9-methoxyluminarine ligand is selectively and effectively quenched by the parallel c-*MYC* G-quadruplex via static mode due to the formation of a 1:1 stoichiometry of the 9-MeLM/c-*MYC* complex. The fluorescence lifetimes calculated for 9-methoxyluminarine (9-MeLM) and its complexes with DNA (9-MeLM/DNA, 1:10 or 1:20) clearly indicated static quenching as there is no apparent change in the fluorescence lifetime [21]. Thus, we have assumed that all the tested parallel G4s quenched 9-methoxyluminarine fluorescence by static interactions forming non-fluorescent complexes. The obtained values of the correlation coefficient indicate that the static quenching fits in the Stern–Volmer model as assumed (Table 2). 

The formation of complex was further confirmed from the values of the quenching rate constant k_q_, which was estimated from the values of K_SV_ and the value of the fluorescence lifetime of 9-MeLM in Tris buffer (τ_0_ = 9.98 ns) [21] in the absence of G4, using the following equation:

k_q_ = K_SV_/τ_0_ (2)

The obtained k_q_ values (collected in Table 2) are higher than the previously reported values for various quenchers in the presence of the biopolymer of 2 × 10^10^ M^−1^s^−1^ (maximum value for scatter collision quenching constant) [42]. Thus, the obtained results indicate that the quenching of 9-methoxyluminarine by c-*MYC* is caused by formation of the complex and is not induced by collision-mediated quenching. 

Using the definition of the association constant and the fact that the fluorescence intensity in the presence of a quencher is proportional to the concentration of uncomplexed fluorophores ([L] = [L_0_] − [LQ]), one can obtain an expression describing the ratio of fluorescence intensities without and in the presence of a quencher:

F_0_/F =1 + K [Q] (3)

In the case of static quenching, the Stern–Volmer quenching constant (K_SV_) from Equation (1) has been replaced by the association constant K in Equation (3). However, one should remember that [Q] in Equation (3) is not the total but a free amount of quencher. Thus, for small [Q] values in the case of complex formation, the Stern–Volmer quenching constant is not equal to K, but rather

K_SV_ = K/(1 + K[L_0_]) (4)

where [L_0_] is initial concentration of fluorophore [43].

Thus, the experimental quenching constant is equal to the equilibrium one (complexing) only if K[L_0_] << 1. In our case, the starting concentration of 9-MeLM was only 1 μM, so we decided to interpret K_SV_ as the association constant, K. The estimated values of the K data along with correlation coefficient are given in Table 2. The calculated K values are a few times higher for c-*MYC1* and c-*MYC2* in comparison with other parallel G4s. Particularly, while analyzing the order of obtained binding constants, K for c-*MYC* with and without flanking sequences, we noticed that the lack of flanking sequences at the 3′-end of c-*MYC* G4 is correlated with a higher value of K. Thus, these results suggest that 9-MeLM preferentially binds to the 3′end of the c-*MYC* promoter.

## 3. Material and Methods

### 3.1. Materials

9-Methoxyluminarine (9-MeLM) was obtained as previously performed [21]. The stock solutions of 9-MeLM were prepared in H_2_O at a concentration of 1.33 mM and was stored at 4 °C. Tris Base (CAS Number 77-86-1) and Tris HCl (CAS Number 1185-53-1) were obtained from Aldrich Chemical Co. (Poznań, Poland) and used as received. All experiments were carried out using quartz cuvettes with a 10 mm optical path, which in the case of fluorescence measurements was for the excitation direction and a 4 mm path length in the emission direction.

### 3.2. Oligonucleotides and Sample Preparation

The G-quadruplex-forming deoxyribonucleotides (Table 3) were purchased from Genomed (Warsaw, Poland) and were used without further purification. Each strand concentration was determined from the absorbance at 260 nm measured at neutral pH and an elevated temperature of 85 °C using extinction coefficients of 254,600 M^−1^cm^−1^ (c-*MYC*), 257,600 M^−1^cm^−1^ (c-*KIT1*), 214,900 M^−1^cm^−1^ (*RET*), 241,800 M^−1^cm^−1^ (catG4), 179,500 M^−1^cm^−1^ (c-*MYC1*), 219,000 M^−1^cm^−1^ (c-*MYC2*), and 219,000 M^−1^cm^−1^ (c-*MYC3*) as calculated from the published values of molar absorptivities of nucleotides [44]. Before use, the oligonucleotide solution was heated at 90 °C for 5 min, allowed to slowly cool to room temperature and then stored at 4 °C overnight. 

### 3.3. Circular Dichroism Spectroscopy (CD)

Circular dichroism spectra were recorded at 25 °C on a Jasco J-810 spectropolarimeter (Jasco, Tokyo, Japan), equipped with a Peltier accessory (Jasco, Kyoto, Japan). Each spectrum represented an average of three scans accumulated with a scan speed of 200 nm/min from 220 to 450 nm. The corresponding buffer (10 mM Tris–HCl with or w/o 100 mM KCl) was used as a blank solution. A spectrum of buffer solution was subtracted from the spectra of the investigated probes. CD spectra were recorded at a strand concentration of 5 μM. The ligand was added to G4 DNA or unfolded oligonucleotide solutions at increasing concentrations from 0.5 to 5 molar equivalents. After each addition of the ligand, the titrated solution was incubated for 3 min prior the CD spectrum measurement. The total volume of added ligand was kept below 2% to avoid dilution. The corresponding UV–vis spectra were collected simultaneously in channel 3, whereas channels 1 and 2 monitored the CD signal and HT (voltage), respectively.

### 3.4. UV Melting Profiles

The melting curves were recorded on a Jasco J-810 spectropolarimeter (Jasco, Tokyo, Japan) equipped with a Jasco Peltier temperature-controlled cell holder. Samples were prepared by mixing the 2 μM oligonucleotide solution in a 10 mM Tris–HCl buffer (pH 7.2) containing 90 mM LiCl and 10 mM KCl immediately prior the experiment. The melting profiles were recorded in both the absence and presence of 3 equiv. of ligand in a 10–90 °C range with a 1 °C/min scan speed. Data were collected at 295 nm as a function of temperature. The determination of melting temperatures was carried out using a method similar to that previously published [45,46]. The melting temperatures (Tm) were determined as the maximum of the first derivative of the melting curves. Each Tm value was an average of three independent measurements.

### 3.5. Fluorescence Spectroscopy

Fluorescence spectra were recorded on a Jasco Spectrofluorimeter (Tokyo, Japan). All fluorescence measurements were performed under the same spectral conditions: the slit width for excitation was 5 nm and that for emission was also 5 nm, and the excitation wavelength was set at 390 nm The emission spectra were collected in a wavelength range of 400–650 nm at constant temperature of 25 °C. The concentration in each experiment was optimized to have absorbance of the ligand < 0.05 at excitation wavelength in order to avoid the inner filter effect. The fluorescence titration experiments were performed at 1 µM concentration of 9-MeLM, by adding increasing amounts of prefolded G4s solutions. After each addition of G4s, the titrated solution was incubated for 3 min prior to the fluorescence spectrum measurement. The final volume of titrant (G4) was kept below 2% to avoid dilution.

## 4. Summary and Conclusions

Here, we presented the results of our study on the interactions between the 9-MeLM ligand and c-*MYC* G-quadruplexes with various lengths, which have a core sequence identical to that of c-*MYC* except for the 5′ and 3′ terminal flanking regions. Particularly, titration of 9-MeLM with c-*MYC1* caused almost 70% quenching of the fluorescence signal that was about twice as strong, compared to that caused by c-*MYC*. The order of the obtained binding constants is as follows: c-*MYC3* (with 3′-end flanking sequence) < c-*MYC* (with both 5′-end and 3′-end flanking sequences) < c-*MYC2* (with 5′-end flanking sequence) < c-*MYC1* (without any flanking sequence). These results indicate that a lack of flanking regions provide better π-stacking possibilities for the planar core of the 9-MeLM ligand. These results also suggest that 9-MeLM preferentially binds to the 3′end of the c-*MYC* promoter; however, this hypothesis should be verified by docking simulations or NMR studies.

The 9-MeLM ligand binds with specificity not only to the parallel-stranded c-*MYC* G-quadruplex but also to other parallel G4s, exhibiting association-quenched emission, as a clear decrease in the fluorescence intensity was observed in all cases. Thus, 9-methoxyluminarine occurred to be an efficient turn-off probe for parallel G4s and can be used as a simple and fast tool to recognize the parallel topology of G-quadruplexes in in vitro conditions or in lab screening assays.

## Figures and Tables

**Table 1:** Melting temperatures (Tm ± SD) of G-quadruplexes’ DNA obtained by monitoring absorbance at 295 nm.

G4 DNA	Tm [°C] ^a^	Tm G4 + L [°C] ^b^
c-*MYC*	79.10 ± 0.10	80.6 ± 1.7
c-*KIT1*	52.9 ± 1.0	54.40 ± 0.60
*RET*	75.30 ± 0.80	76.4 ± 1.2
catG4	63.30 ± 0.10	62.80 ± 0.30

^a^ T_m_ of G4s DNA in the presence of 10 mM KCl and 90 mM LiCl in 10 mM Tris–HCl buffer (pH 7.2). ^b^ T_m_ of G4s DNA incubated with 3 equiv. of ligand. Data were collected at 295 nm. Typically, three replicate experiments were performed.

**Table 2:** Parameters for the interaction of 9-methoxyluminarine with parallel G-quadruplexes determined using the S-V model in fluorescence titration experiments (K_SV_—Stern–Volmer quenching constant is interpreted as K—binding constant, k_q_—quenching rate constant, R—correlation coefficient, λex = 390 nm).

G4	K (×10^4^ M^−1^)	k_q_ (×10^12^ M^−1^s^−1^)	R
*RET*	3.0 ± 0.3	3	0.994
c-*KIT1*	3.7 ± 0.6	4	0.990
catG4	5.3 ± 0.1	5	0.999
c-*MYC*	5.9 ± 0.7	6	0.999
c-*MYC1*	20 ± 4	20	0.997
c-*MYC2*	17 ± 3	17	0.999
c-*MYC3*	4.5 ± 0.5	5	0.990

**Table 3:** The oligonucleotides used in this study and their PDB ID. The guanines engaged in G-tetrad formation are shown in **bold**, whereas bases involved in sidewide loops are shown in *italics*.

G4	Sequence 5′-3′	PDB ID
*RET*	**GGG***GCG***GGG***C***GGG***GCG***GGG**T	2L88
c-*KIT1*	A**GGG***A***GGG***C*GC*T***GGG***AGGAG***GG**	2O3M
catG4	T**GGG***TA***GGG***C***GGG***TT***GGG**AAA	-
c-*MYC*	TGA**GGG***T***GGG***TA***GGG***T***GGG**TAA	2L7V
c-*MYC1*	**GGG** *T* **GGG** *TA* **GGG** *T* **GGG**	-
c-*MYC2*	TAA**GGG***T***GGG***TA***GGG***T***GGG**	-
c-*MYC3*	**GGG***T***GGG***TA***GGG***T***GGG**TAA	-

## Data Availability

All data are provided in this article.

## Supplementary Materials

The following supporting information can be downloaded at https://www.mdpi.com/article/10.3390/molecules29163907/s1. Figure S1: UV–vis titration spectra of G-quadruplexes c-*KIT1* (A), *RET* (B) and catG4 (C) with increasing amounts of 9-methoxyluminarine ligand. Conditions: 10 mM Tris–HCl buffer (pH 7.2), 100 mM KCl, [G4] = 5 µM; Figure S2: Normalized UV melting profiles of *RET* (A), c-*MYC* (B), c-*KIT1* (C) and catG4 (D) of G4 oligonucleotides (black 10–90 °C, red 90–10 °C) and oligonucleotides in the presence of 9-methoxyluminarine ligand (3 equiv.) (green 10–90 °C, yellow 90–10 °C). Melting profiles were recorded at 295 nm, in 10–90 °C range, 1 °C/min heating/cooling rate. Buffer conditions: 10 mM KCl and 90 mM LiCl in 10 mM Tris–HCl buffer (pH 7.2); Figure S3: Fluorescence titration spectra of 9-methoxyluminarine ligand (1 µM) with G4 *RET* (A), G4 c-*MYC* (B), G4 c-*KIT1* (C) and G4 catG4 (D) (0–25 µM) in Tris–HCl buffer (10 mM, pH 7.2) containing 100 mM KCl; Figure S4: Fluorescence quenching Stern–Volmer plot of 9-methoxyluminarine (9-MeLM) with increasing concentration of G4 *RET* (A), G4 c-*KIT1* (B) and G4 catG4 (C) in Tris–HCl buffer (10 mM, pH 7.2) containing 100 mM KCl; Figure S5: Fluorescence quenching Stern–Volmer plot of 9-methoxyluminarine (9-MeLM) with increasing concentration of G4 c-*MYC* (A), c-*MYC1* (B), G4 c-*MYC2* (C), c-*MYC3* (D) in Tris–HCl buffer (10 mM, pH 7.2) containing 100 mM KCl.
