# Supplementary material for: Fluorescence Turn-Off Ligand for Parallel G-Quadruplexes
Source: Molecules. 2024 Aug 18;29(16):3907. doi: 10.3390/molecules29163907 (PMC11357100; doi:10.3390/molecules29163907)
Supplement: Supplementary file 1 [file molecules-29-03907-s001.zip › molecules-3119805-supplementary.pdf]

Supporting Information

Fluorescence Turn-Off Ligand for Parallel G-Quadruplexes

Joanna Nowak-Karnowska [1], Agata Głuszyńska [1], Joanna Kosman [1,2] and Anna Dembska [1,*]

[a] Department of Bioanalytical Chemistry, Faculty of Chemistry, Adam Mickiewicz University, Uniwersytetu Poznańskiego 8, Poznań 61-614, Poland;

[b] Laboratory of Molecular Assays and Imaging, Institute of Bioorganic Chemistry, Polish Academy of Sciences, Noskowskiego 12/14, 61-704 Poznań, Poland;

[*] Correspondence: aniojka@amu.edu.pl (A.D)

Contents:

Figure S1. UV–vis titration spectra of G-quadruplexes c-*KIT1* (A), *RET* (B) and catG4 (C) with increasing amounts of 9-methoxyluminarine ligand. Conditions: 10 mM Tris–HCl buffer (pH 7.2), 100 mM KCl, [G4] = 5 μM.

Figure S2. Normalized UV melting profiles of *RET* (A), c-*MYC* (B), c-*KIT1* (C) and catG4 (D) of G4 oligonucleotides (black 10–90°C, red 90–10°C) and oligonucleotides in the presence of 9-methoxyluminarine ligand (3 equiv.) (green 10–90°C, yellow 90–10°C). Melting profiles were recorded at 295 nm, in 10–90°C range, 1°C/min heating/cooling rate. Buffer conditions: 10 mM KCl and 90 mM LiCl in 10 mM Tris–HCl buffer (pH 7.2).

Figure S3. Fluorescence titration spectra of 9-methoxyluminarine ligand (1 μM) with G4 *RET* (A), G4 c-*MYC* (B), G4 c-*KIT1* (C) and G4 catG4 (D) (0–25 μM) in Tris–HCl buffer (10 mM, pH 7.2) containing 100 mM KCl.

Figure S4. Fluorescence quenching Stern–Volmer plot of 9-methoxyluminarine (9-MeLM) with increasing concentration of G4 *RET* (A), G4 c-*KIT1* (B) and G4 catG4 (C) in Tris–HCl buffer (10 mM, pH 7.2) containing 100 mM KCl.

Figure S5. Fluorescence quenching Stern–Volmer plot of 9-methoxyluminarine (9-MeLM) with increasing concentration of G4 c-*MYC* (A), c-*MYC1* (B), G4 c-*MYC2* (C), c-*MYC3* (D) in Tris–HCl buffer (10 mM, pH 7.2) containing 100 mM KCl.

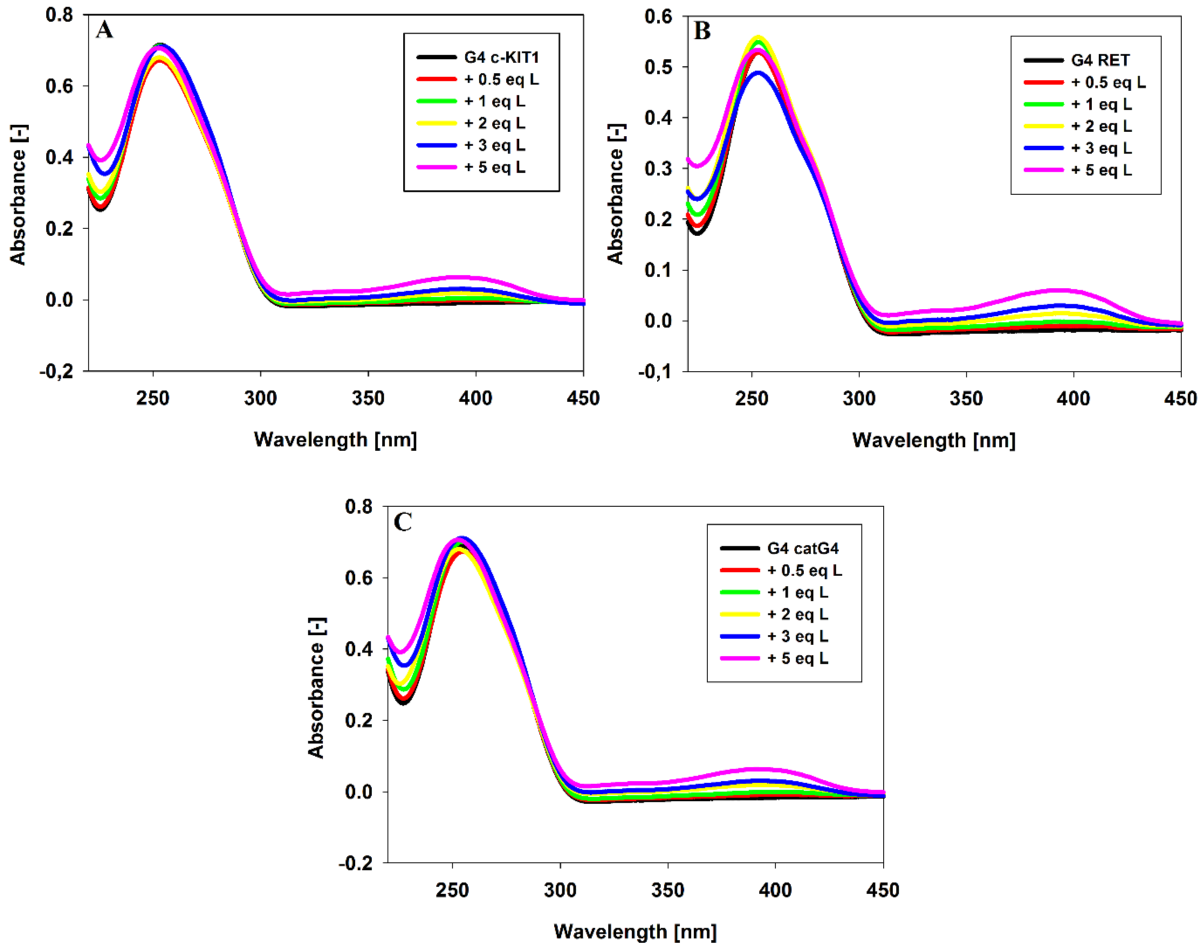

Figure S1. UV–vis titration spectra of G-quadruplexes c-*KIT1* (A), *RET* (B) and catG4 (C) with increasing amounts of 9-methoxyluminarine ligand. Conditions: 10 mM Tris–HCl buffer (pH 7.2), 100 mM KCl, [G4] = 5 μM.

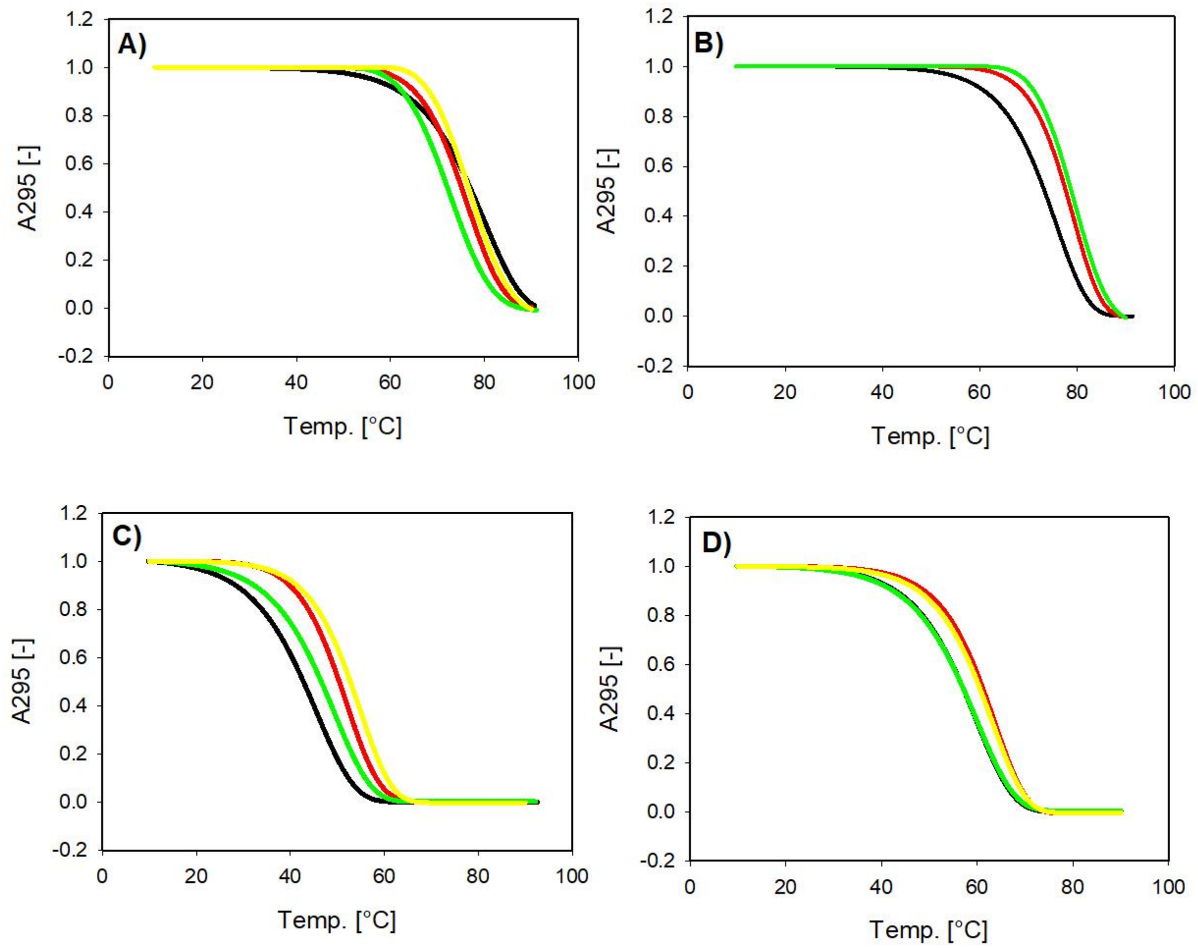

Figure S2. Normalized UV melting profiles of *RET* (A), c-*MYC* (B), c-*KIT1* (C) and catG4 (D) of G4 oligonucleotides (black 10–90°C, red 90–10°C) and oligonucleotides in the presence of 9-methoxyluminarine ligand (3 equiv.) (green 10–90°C, yellow 90–10°C). Melting profiles were recorded at 295 nm, in 10–90°C range, 1°C/min heating/cooling rate. Buffer conditions: 10 mM KCl and 90 mM LiCl in 10 mM Tris–HCl buffer (pH 7.2).

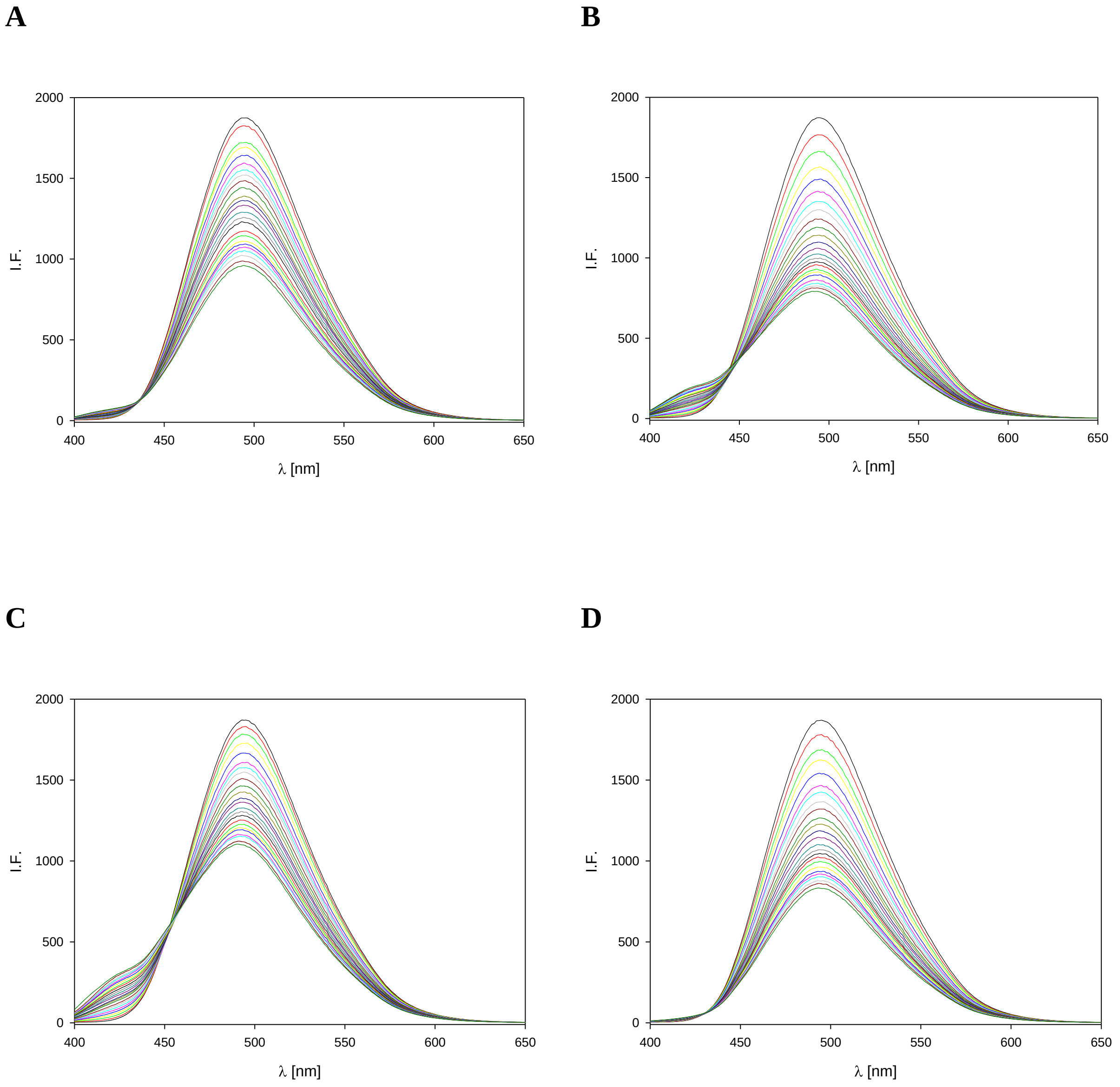

Figure S3. Fluorescence titration spectra of 9-methoxyluminarine ligand (1 μM) with G4 *RET* (A), G4 c-*MYC* (B), G4 c-*KIT1* (C) and catG4 (D) (0–25 μM) in Tris–HCl buffer (10 mM, pH 7.2) containing 100 mM KCl.

A

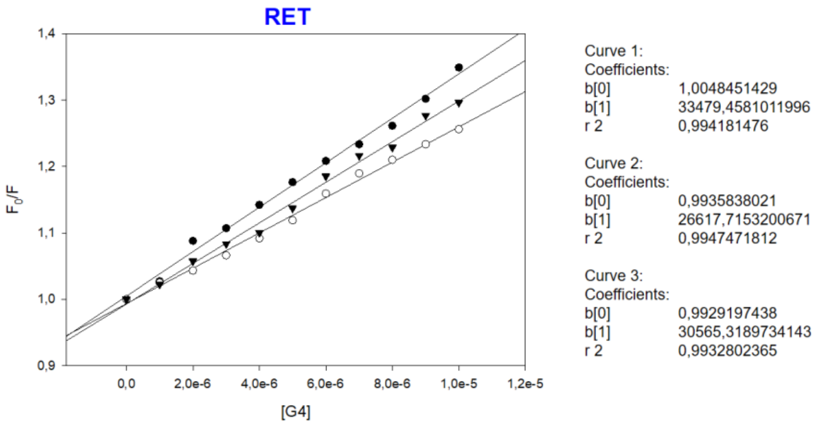

B

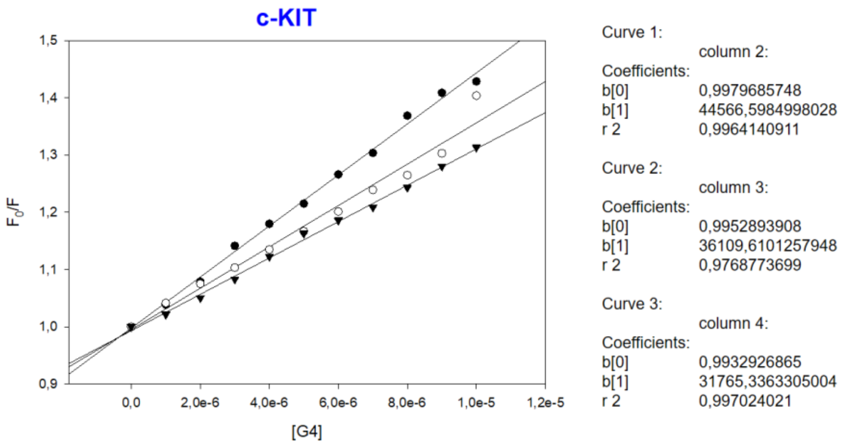

C

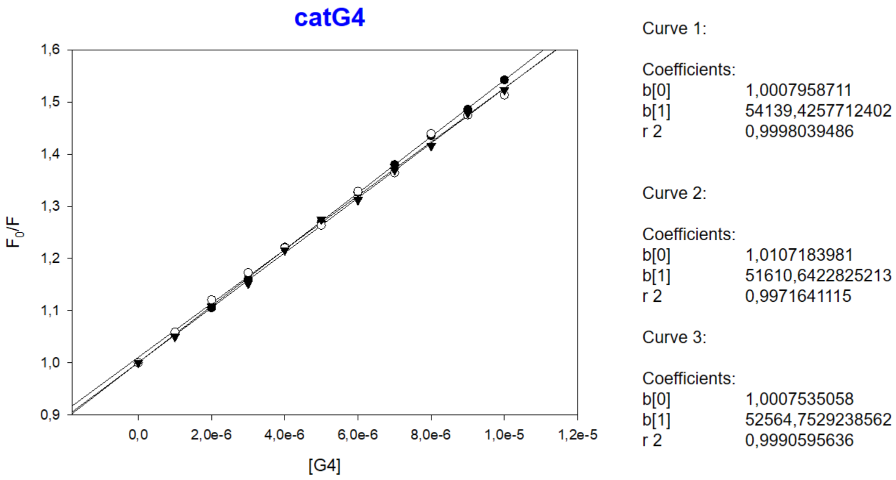

Figure S4. Fluorescence quenching Stern–Volmer plot of 9-methoxyluminarine (9-MeLM) with increasing concentration of G-quadruplexes *RET* (A), G4 c-*KIT1* (B) and G4 catG4 (C) in Tris–HCl buffer (10 mM, pH 7.2) containing 100 mM KCl.

A

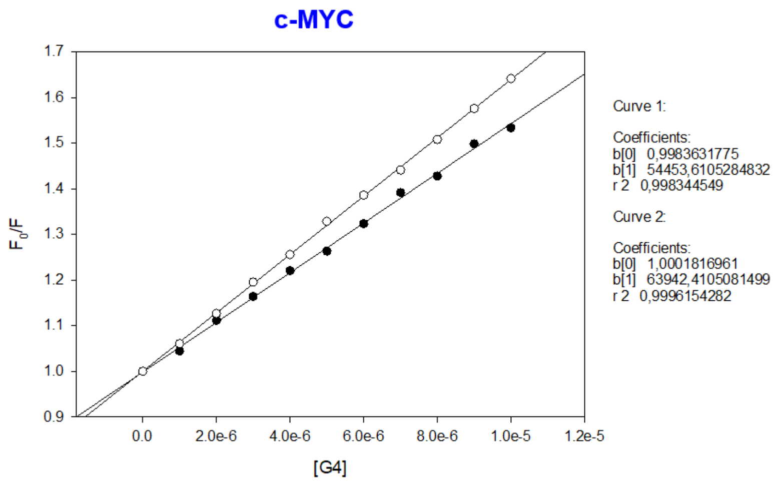
c-MYC
1.7
1.6
1.5
1.4
1.3
1.2
1.1
1.0
0.9
F_0/F
0.0
2.0e-6
4.0e-6
6.0e-6
8.0e-6
1.0e-5
1.2e-5
[G4]
Curve 1:
Coefficients:
b[0] 0,9983631775
b[1] 54453,6105284832
r 2 0,998344549
Curve 2:
Coefficients:
b[0] 1,0001816961
b[1] 63942,4105081499
r 2 0,9996154282

B

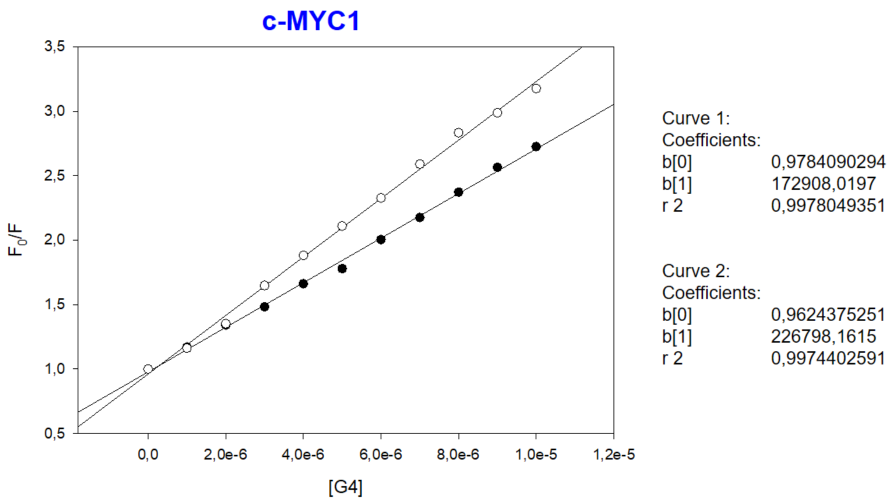
c-MYC1
3,5
3,0
2,5
2,0
1,5
1,0
0,5
F_0/F
0,0
2,0e-6
4,0e-6
6,0e-6
8,0e-6
1,0e-5
1,2e-5
[G4]
Curve 1:
Coefficients:
b[0] 0,9784090294
b[1] 172908,0197
r 2 0,9978049351
Curve 2:
Coefficients:
b[0] 0,9624375251
b[1] 226798,1615
r 2 0,9974402591

C

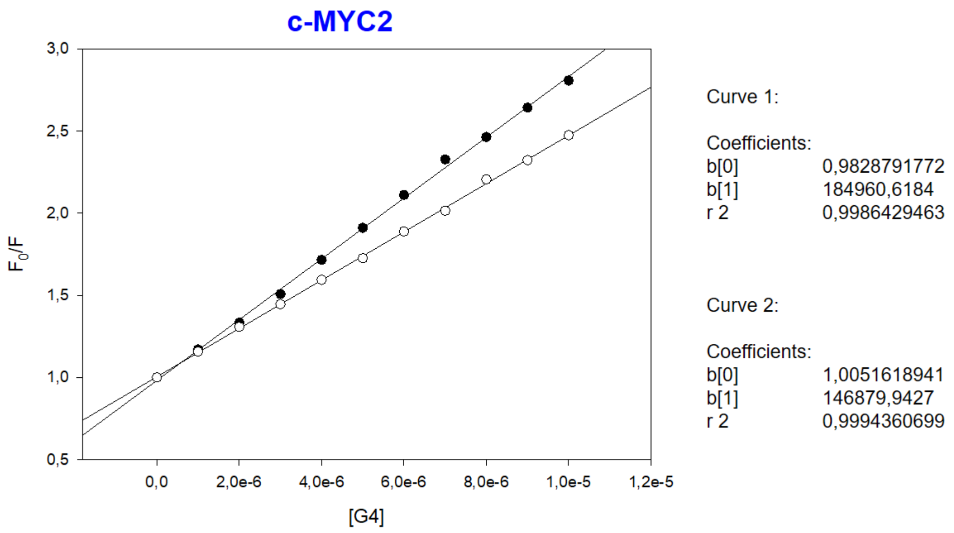
c-MYC2
3,0
2,5
2,0
1,5
1,0
0,5
F_0/F
0,0
2,0e-6
4,0e-6
6,0e-6
8,0e-6
1,0e-5
1,2e-5
[G4]
Curve 1:
Coefficients:
b[0] 0,9828791772
b[1] 184960,6184
r 2 0,9986429463
Curve 2:
Coefficients:
b[0] 1,0051618941
b[1] 146879,9427
r 2 0,9994360699

D

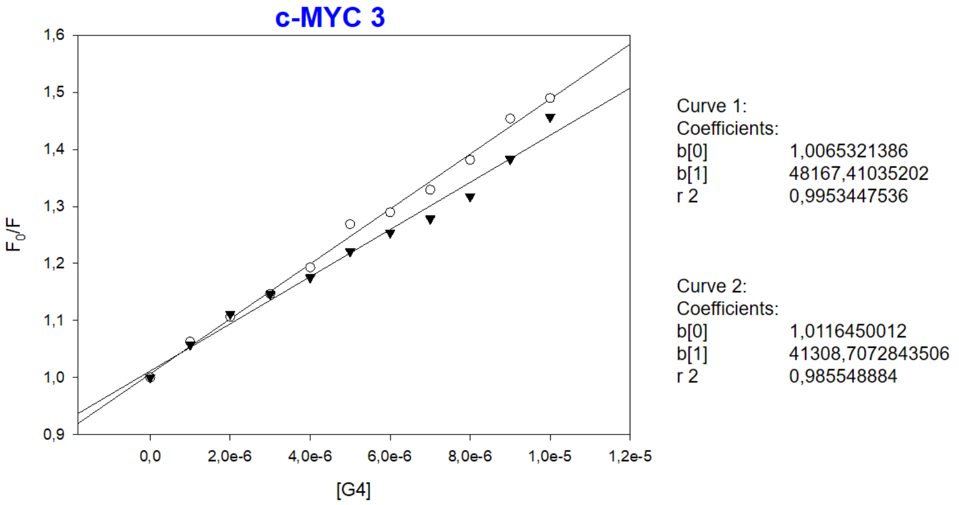

Figure S5. Fluorescence quenching Stern–Volmer plot of 9-methoxyluminarine (9-MeLM) with increasing concentration of with G-quadruplexes c-*MYC* (A), c-*MYC1* (B), c-*MYC2* (C), c-*MYC3* (D) in Tris–HCl buffer (10 mM, pH 7.2) containing 100 mM KCl.
